# Somatic molecular subtyping of prostate tumors from *HOXB13* G84E carriers

**DOI:** 10.18632/oncotarget.15196

**Published:** 2017-02-08

**Authors:** Tamara L. Lotan, Alba Torres, Miao Zhang, Jeffrey J. Tosoian, Liana B. Guedes, Helen Fedor, Jessica Hicks, Charles M. Ewing, Sarah D. Isaacs, Dorhyun Johng, Angelo M. De Marzo, William B. Isaacs

**Affiliations:** ^1^ Department of Pathology, Johns Hopkins University School of Medicine, Baltimore, MD, USA; ^2^ Department of Oncology, Johns Hopkins University School of Medicine, Baltimore, MD, USA; ^3^ Department of Pathology the University of Texas MD Anderson Cancer Center, Houston, TX, USA; ^4^ Brady Urological Institute, Johns Hopkins University School of Medicine, Baltimore, MD, USA

**Keywords:** prostatic adenocarcinoma, HOXB13 G84E, familial prostate cancer, PTEN, ERG

## Abstract

A recurrent germline mutation (G84E) in the *HOXB13* gene is associated with early onset and family history-positive prostate cancer in patients of European descent, occurring in up to 5% of prostate cancer families. To date, the molecular features of prostate tumors occurring in *HOXB13* G84E carriers have not been studied in a large cohort of patients. We identified 101 heterozygous carriers of G84E who underwent radical prostatectomy for prostate cancer between 1985 and 2011 and matched these men by race, age and tumor grade to 99 *HOXB13* wild-type controls. Immunostaining for HOXB13, PTEN, ERG, p53 and SPINK1 as well as RNA *in situ* hybridization for ETV1/4/5 were performed using genetically validated assays. Tumors from G84E carriers generally expressed HOXB13 protein at a level comparable to benign and wild-type glands. *ETS* gene expression (either *ERG* or *ETV1/4/5*) was seen in 36% (36/101) of tumors from G84E carriers compared to 68% (65/96) of the controls (*p* < 0.0001). PTEN was lost in 11% (11/101) of G84E carriers compared to 25% (25/99) of the controls (*p* = 0.014). PTEN loss was enriched among ERG-positive compared to ERG-negative tumors in both groups of patients. Nuclear accumulation of the p53 protein, indicative of underlying *TP53* missense mutations, was uncommon in both groups, occurring in 1% (1/101) of the G84E carriers versus 2% (2/92) of the controls (*p* = NS). Taken together, these data suggest that genes other than *ERG* and *PTEN* may drive carcinogenesis/progression in the majority of men with germline *HOXB13* mutations.

## INTRODUCTION

A significant proportion of prostate cancer risk is inherited and the genetic variations modulating this risk are increasingly well known [1, 2]. A rare mutation in the *HOXB13* gene, G84E, is reproducibly associated with a 4- to 8-fold increased risk of prostate cancer [3–12]. This mutation occurs in ~1% of unselected PCa cases, a frequency which rises to over 3% in men with early onset, family history positive disease and ~5% in prostate cancer families. It is most prevalent in populations with Nordic ancestry [3–12]. *HOXB13* is a homeobox transcription factor demonstrated to play a role in prostate development and in conferring androgen responsiveness of prostate specific gene expression [13, 14]. However the mechanism by which the G84E mutation contributes to prostate cancer risk remains unknown and it is unclear whether prostate tumors arising in G84E carriers are molecularly distinct from those occurring in the general population.

Prostate cancer is a clinically and molecularly heterogeneous disease, and the common molecular subtypes of primary prostate cancer are well known from numerous whole exome sequencing efforts [15]. Among the most common somatic genomic alterations in prostate cancer are rearrangements involving *ETS*-family transcription factors, of which *ERG* is the most common, seen in nearly half of all prostate tumors [16, 17]. Less commonly, rearrangements or over-expression of *ETV1*, *ETV4* and *ETV5* occurs, and these alterations are typically mutually exclusive with one another and with *ERG* rearrangement, suggesting functional redundancy [15, 16, 18]. Interestingly, *ERG* rearrangement prevalence varies significantly by racial ancestry [19–24], suggesting that germline genetics and/or environmental factors could affect the frequency of somatic genomic alterations in prostate cancer. PTEN is the most commonly inactivated tumor suppressor gene in prostate cancer, and the prevalence of *PTEN* deletion increases dramatically with increasing tumor grade and stage [25–27]. Unlike *ERG* rearrangements [17], PTEN loss is reproducibly associated with worse oncologic outcomes, including biochemical recurrence, metastasis and death from prostate cancer [26–29]. PTEN loss is more common in tumors with *ERG* rearrangements than those without and likely occurs subsequent to *ERG* alteration in most cases [30–33].

Because the prevalence is low, few prior studies have examined the molecular and pathologic features of prostate tumors arising in G84E carriers. In the largest study of nearly 10,000 men undergoing radical prostatectomy at Johns Hopkins University and the University of Michigan, patients with the G84E variant were younger at the time of diagnosis and more likely to have a family history of prostate cancer compared to non-carrier controls from the same time period [34]. However carrier status was not associated with Gleason grade or pathologic tumor stage in this cohort, though other studies have conflicting associations [35, 36]. In a follow-up study using the University of Michigan cohort, the prevalence of two molecular subtypes of prostate cancer was assessed among 23 prostate tumors from G84E carriers [37]. The prevalence of ERG-positive tumors was lower and the prevalence of SPINK1-positive tumors was higher in G84E carriers than rates reported in most historical surgical cohorts unselected for *HOXB13* status, suggesting the potential for significant molecular differences between tumors from G84E carriers and non-carriers. However, the numbers of cases assessed in this study were small and matched control non-carriers were not studied.

Here, we provide a follow-up molecular study on prostate tumors from 101 G84E carriers who underwent radical prostatectomy at Johns Hopkins with age, race and grade-matched control non-carriers. Using genetically validated assays, we confirm that the prevalence of ETS expression is significantly lower among carriers compared to non-carriers, and further demonstrate that PTEN loss, a genomic alteration associated with *ERG* rearrangement and poor outcomes, is also significantly less common in this population. These findings suggest that genes other than *ERG* and *PTEN* may drive carcinogenesis/progression in the majority of men with germline *HOXB13* mutations.

## RESULTS

### Expression of HOXB13 protein among G84E carriers and non-carriers

**Figure 1:**
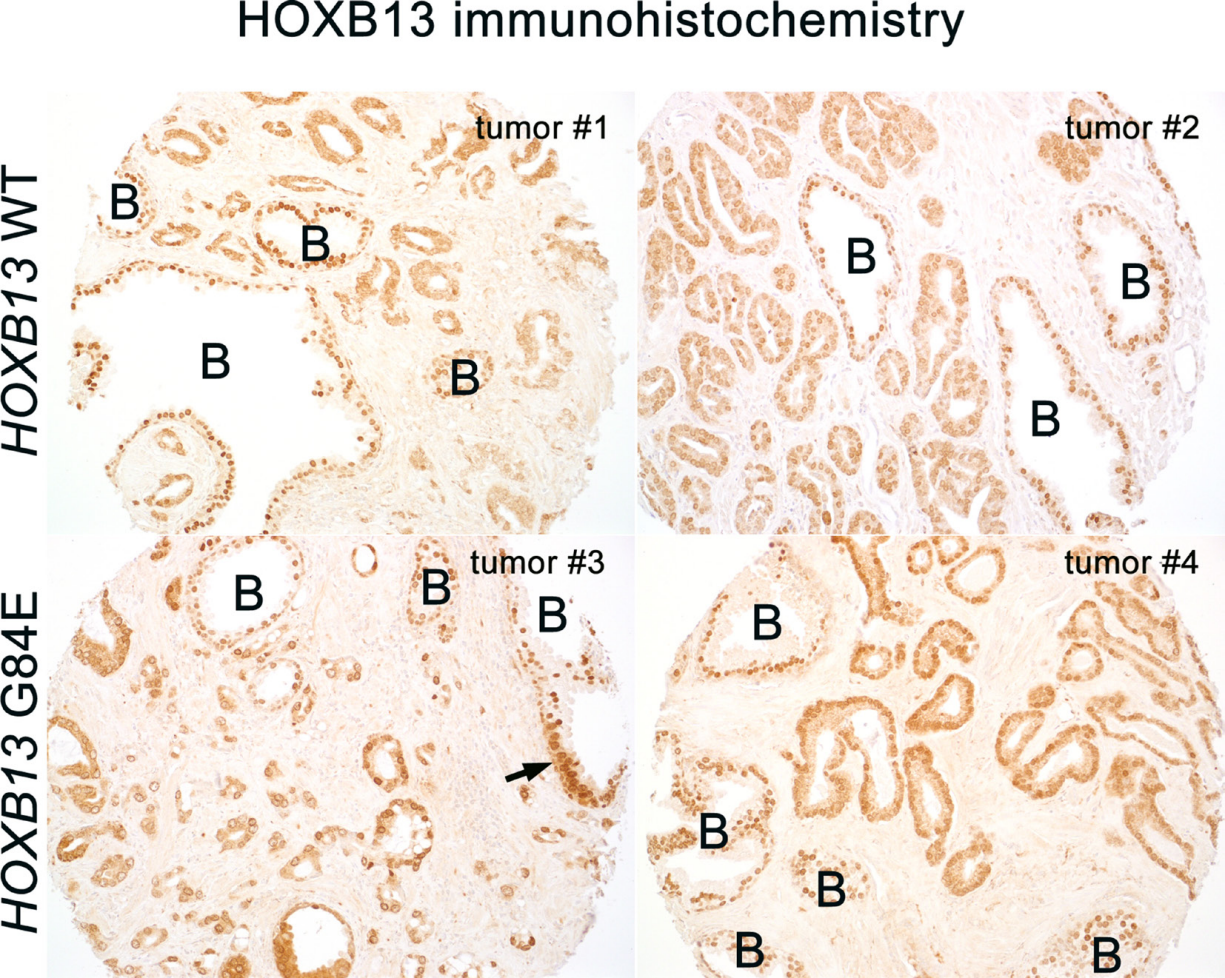
HOXB13 protein is expressed in the nuclei of benign glands (denoted by a B) at similar levels in G84E carriers and non-carriers (G84E WT) In tumor cells, cytoplasmic HOXB13 levels are somewhat higher than that seen in benign glands in both carriers and non-carriers, however some variability is noted. Nuclear levels in carrier tumors are similar or higher to that seen in non-carriers. Arrow indicates a focal region of prostatic intraepithelial neoplasia (PIN) with increased cytoplasmic HOXB13 expression compared to the surrounding benign glandular cells. Images at 200× magnification.

A total of 101 G84E carriers (95% or 101/106) with full pathologic data and immunostaining results were matched using stratified random sampling by age and Gleason score to European-American genotyped non-carrier patients from one of three tissue microarrays (TMA) previously constructed at Johns Hopkins without selection for prostate cancer oncologic outcomes (see Methods). Clinical and pathologic data are shown in Table 1. G84E carriers had smaller prostates compared to non-carriers (47 vs 50 grams, *p* = 0.004), but were otherwise well matched for Gleason grade and stage with non-carriers. HOXB13 was generally diffusely expressed in the nuclei of benign prostate glands from G84E carriers at a level qualitatively comparable to that seen in non-carriers (Figure 1). Among both carriers and non-carriers, HOXB13 protein expression was generally a bit higher in the cytoplasm of tumor cells compared to benign glands. HOXB13 was present in the nuclei of most tumor glands in both carriers and non-carriers. Though there was some variability in intensity, carriers had nuclear levels at least as high, if not higher, than non-carriers in most cases by qualitative analysis (Figure 1). Collectively, these data indicated that protein expression of the transcription factor was not compromised by the missense mutation.

**Table 1:** Clinical-pathologic and molecular features of HOXB13 cases and controls

	*HOXB13* WT (*n* = 99)	*HOXB13* G84E (*n* = 101)	*P*-value
**Year of surgery (median, IQR)**	2001 (2001–2001)	2004 (2001–2007)	< 0.001
**Age (median, IQR)**	55 (51–60)	55 (51–61)	0.8
**PSA, ng/ml**	5.5 (4.4–7.9)	5.5 (4.0–8.2)	0.8
**Prostate weight, g**	50 (44–61)	47 (39–53)	0.01
**RP grade group** ** 1 (GS6)** ** 2 (GS3+4=7)** ** 3 (GS4+3=7)** ** 4 (GS8)** ** 5 (GS9-10)**	57 (57.6%) 25 (25.3%) 9 (9.1%) 3 (3.0%) 5 (5.1%)	57 (56.4%) 27 (26.7%) 10 (9.9%) 2 (3.0%) 5 (5.0%)	0.99
**RP pathologic stage** ** pT2N0** ** pT3aN0** ** pT3bN0** ** pTxN1**	69 (69.7%) 22 (22.2%) 5 (5.1%) 3 (3.0%)	69 (68.3%) 25 (24.7%) 3 (3.0%) 4 (4.0%)	0.8
**ERG+**	56 (56.6%)	33 (33%)	0.001
**ETV1+**	7 (7.6%)	4 (4.0%)	0.4
**ETV4+**	2 (2.2%)	1 (1.0%)	0.9
**ETV5+**	0 (0.0%)	0 (0.0%)	NA
**ETS+**	65 (67.7%)	36 (35.6%)	< 0.0001
**PTEN loss**	25 (25.3%)	11 (10.9%)	0.01
**Genotype** ** PTEN+ ETS-** **PTEN+ ETS+** ** PTEN- ETS+** ** PTEN- ETS-**	30 (31.3%) 40 (41.7%) 24 (25.0%) 2 (2.1%)	64 (63.3%) 26 (25.7%) 10 (9.9%) 1 (1.0%)	< 0.0001
**SPINK1+**	6 (6.5%)	9 (8.9%)	0.7
**Nuclear p53 accumulation**	2 (2.0%)	1 (1.0%)	0.9

### Association of G84E carrier status with tumor *ETS*, *PTEN*, *SPINK1* and *TP53* status

**Figure 2:**
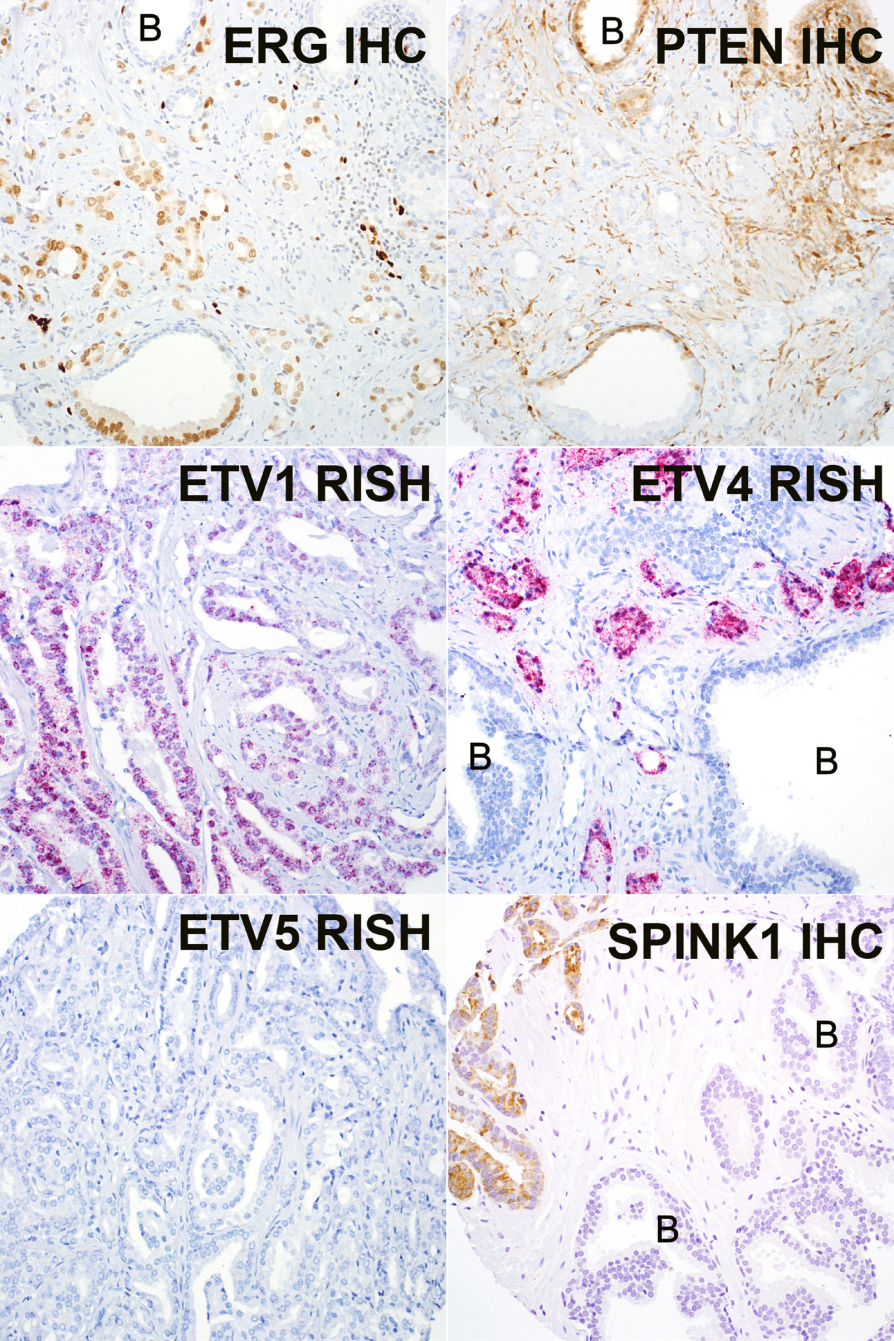
Representative images of ERG, PTEN, and SPINK1 immunohistochemistry (IHC) and ETV1/4/5 RNA *in situ* hybridization (RISH) ERG and PTEN immunostaining are shown from the same case, which expresses ERG (nuclear brown) and has lost PTEN expression in tumor cells, with retained expression in neighboring benign glands (denoted by a B). ETV1 and ETV4 RISH are from two representative positive cases (granular red staining) and are expressed in tumor glands, but not in intermingled benign glands (denoted by a B). ETV5 was negative in all cases. SPINK1 IHC is positive in a representative tumor case and negative in nearby benign glands (denoted by a B). Images at 200x magnification.

The prevalence of ERG expression among tumors from G84E carriers was significantly lower than that seen in non-carriers (33% or 33/101 vs 57% or 56/99; *p* = 0.001) (Table 1, Figure 2). A similar trend was seen for ETV1 (4% in *HOXB13* G84E vs. 8% in *HOXB13* WT) and ETV4 (1% in *HOXB13* G84E vs. 2% in *HOXB13* WT) prevalence, though these did not reach statistical significance due to low frequency of events. ETV5 expression was not observed in any cases in either cohort. Only two cases were identified that co-expressed two ETS genes (both ERG and ETV1, and both in G84E carriers). Although ERG and ETV1 were assayed on separate TMA slides, at least one of these two tumors expressed ERG and ETV1 expression in apparently separate subsets of tumor cells, suggesting a potential collision between two independent clones (Supplementary Figure 2).

PTEN loss was present in only 11% (11/101) of tumors from G84E carriers versus 25% (25/99) of tumors from non-carriers (*p* = 0.014). PTEN loss was more common among ETS-positive compared to ETS-negative tumors in both groups of patients (Table 1). SPINK1 expression was observed in 9% (9/101) of G84E carriers compared to 6% (6/92) of non-carriers, though this difference did not reach statistical significance. SPINK1 expression was generally mutually exclusive with *ETS* gene fusions among G84E carriers as previously reported [38, 39], however 2 cases among non-carriers expressed both SPINK1 and ERG. Finally, nuclear accumulation of the p53 protein, indicative of underlying *TP53* missense mutations, was uncommon in both groups, occurring in 1% (1/101) of the G84E carriers versus 2% (2/92) of the controls (*p* = NS).

## DISCUSSION

The common somatic molecular subtypes of prostate cancer are well known and the germline genetic variants associated with increased risk of prostate cancer are increasingly understood. However, to date, few studies have performed germline-somatic correlations, examining the association between germline variants and tumor somatic genomic alterations. Studies comparing the relative prevalence of molecular subtypes between two populations must be carefully designed since the prevalence of known subtypes can vary by tumor grade and stage, as well as by patient ancestry. Here, we compared the prevalence of several of the most common molecular subtypes of prostate cancer between carriers and non-carriers of a rare germline variant associated with increased prostate cancer risk. Using non-carriers matched to carriers by age, race and tumor grade, we found that the relative prevalence of both ETS and PTEN alterations in 101 *HOXB13* G84E carriers is significantly lower than that seen in non-carriers undergoing surgical treatment at our institution. Though there was a slight increase in the prevalence of SPINK1 positive tumors among G83E carriers compared to non-carriers, this difference did not reach statistical significance. p53 nuclear accumulation, indicative of underlying *TP53* missense mutation, was rare in both groups, not unexpectedly given the relatively low stage and grade of the tumors studied in these cohorts.

The lower rate of *ETS* gene fusions among *HOXB13* G84E carriers compared to non-carriers in the current study reinforces results from an earlier study of 23 G84E carriers at the University of Michigan [37]. This study found that ERG was positive in the dominant tumor nodule in 22% of cases. Though no matched non-carrier controls were available from this previous study, the authors noted that the prevalence of ERG positivity was markedly lower than that reported in prior studies of the general population. In the current study, we found a somewhat higher rate of ERG expression among carriers (33%), however it remained significantly lower (nearly half as common) as the prevalence among non-carriers, and a similar trend was seen for ETV1 and ETV4. Interestingly, the prevalence of SPINK1 expression in the prior University of Michigan study was nearly 30% among G84E carriers. SPINK1 is an alteration that is generally mutually exclusive with *ERG* rearrangement and commonly associated with SPOP gene mutation [39]. In the current study we were not able to confirm an increased prevalence of SPINK1 positivity among carriers (though we did see a slight trend in this direction) and our rates of SPINK1 positivity among carriers were considerably lower (only 9%). However, this discrepancy may be due in part to the fact that the current study examined SPINK1 expression in tissue microarrays (TMAs) whereas the previous study utilized standard sections. SPINK1 expression is frequently heterogeneous, and thus may have been missed in the current study in some cases due to under-sampling.

It remains unclear why *ETS* alterations are so much less common among *HOXB13* G84E carriers compared to matched non-carriers. Previous studies have suggested that the mechanism of *ETS* gene rearrangement may be mediated by androgen signaling in prostate cancer [40], thus it is tempting to conjecture that androgen signaling may be altered in G84E carriers. Norris et al. have demonstrated a strong dependence of the expression of various androgen-responsive genes in multiple prostate cancer cell lines on HOXB13 [14]. However, there is currently no evidence that this HOXB13 influence on AR signaling is impacted by the G84E mutation. Indeed, in the current study, we showed that the mutant protein is expressed in the nuclei in tumors from G84E carriers at levels comparable to that seen in benign glands as well as in glands from non-carriers; however additional studies are necessary to determine how the mutation alters the protein's function *in vivo*.

An interesting finding in the current study is that the lower rate of ETS-positive cases among *HOXB13* G84E carriers compared to matched controls was paralleled by a lower rate of PTEN loss in the carrier group. PTEN is the most commonly lost tumor suppressor in prostate cancer and its loss is uniformly associated with poor outcomes and higher tumor grade and stage in prostate cancer across studies [26–29]. Thus, the finding that PTEN loss is half as common among G84E carriers compared to grade and stage-matched non-carriers is intriguing. Given the tight association of PTEN loss with adverse outcomes in prostate cancer [26–29], this finding would seem to suggest that *HOXB13* G84E carriers may have more indolent disease compared to non-carriers. Previously [34], we observed a nearly two-fold increase in the odds of early-onset disease associated with being a G84E carrier as well as a ~1.5 fold increase in the likelihood of having a family history of prostate cancer. (OR = 1.90; 95% CI = 1.23–2.94; OR = 1.45; 95% 1.32–1.59 respectively). However, there was no effect of carrier status on the proportion of patients with high-grade tumors or with advanced stage (≥ pT3a) tumors by carrier status. Other studies have had mixed results, with some reporting more aggressive features in men with clinically localized G84E positive prostate cancer in Danish and Finnish cohorts [35, 36].

Indeed, interpretation of the finding of less frequent PTEN loss among carriers may also be complicated by tumor ETS status. PTEN loss is two- to five-times more frequent among ERG-positive compared to ERG-negative tumors and we and other groups have reported that PTEN loss occurs subsequent to ERG rearrangement in the majority of prostate tumors [30–33]. Accordingly, *ERG* rearrangement may modify the association of PTEN loss with poor outcome. Though PTEN loss is associated with an increased risk of biochemical recurrence regardless of ERG status [29, 41], when prostate cancer specific death is used as an outcome, ERG-negative tumors with PTEN loss appear to have the highest risk [27, 42]. In the current study, the prevalence of PTEN loss and ETS expression were decreased proportionately among G84E carriers, such that PTEN loss remained enriched among ETS-positive tumors in both carriers and non-carriers. Of interest, we recently reported similar findings in patients of African-American compared to European-American ancestry [22], providing further evidence that 1) germline genetics may be associated with the prevalence of common molecular subtypes of prostate cancer and 2) PTEN and ERG alterations are tightly coupled in primary prostate cancer. Given that the low rate of ERG rearrangement among patients with African-American ancestry may be associated, in part, with the potentially higher rate of anterior-dominant index tumors in this population (tumors known to show less frequent *ERG* rearrangement), it is tempting to speculate that something similar may be occurring among G84E carriers [43, 44]. Among the 78 cases where we could examine the index tumor location, 33% were centered anterior to the urethra, which is somewhat higher than the ~15% rate of anterior-dominant tumors reported in other radical prostatectomy cohorts [45], and perhaps in line with the lower rate of ERG expression among the G84E carriers. However interpretation of these data requires caution as differing tumor locations may largely reflect differing screening protocols among cases and controls. Indeed, G84E carriers often have a strong family history of prostate cancer, and because of this are frequently subjected to increased prostate cancer screening which bias towards detection of more frequent anterior-dominant tumors.

Though this is the largest and most comprehensive study of prostate tumors arising in *HOXB13* G84E carriers, and the only study with matched controls, this work has some limitations that bear discussion. First, this remains a single institution cohort and thus requires independent validation. Second, cases and controls were matched by stratified random sampling of existing tissue microarray cohorts at Johns Hopkins on a limited set of variables. Thus, while age, race, PSA, grade and stage are well matched between cases and controls, there are some notable differences between groups, including prostate weight and year of surgery. Though it is unlikely that these variables are associated with molecular subtypes and would have confounded the results, these differences in follow-up time do exclude the possibility of comparing surgical outcomes between the two groups, another weakness of this study. Another potential limitation is that molecular subtyping was done using tissue microarray samples of the index tumor, which may lead to errors in prevalence estimates for alterations that are heterogeneous, such as PTEN and SPINK1. Future studies may utilize standard tissue sections to mitigate this weakness. In addition, it should be noted that the markers used in this study, such as ERG and PTEN immunohistochemistry, were largely surrogate markers of genomic alterations. Although we have demonstrated that our assays are highly concordant with genomic rearrangement and deletion status of ERG and PTEN, respectively, no surrogate marker is perfect [46, 47]. Finally, it is important to point out that the current study only assesses associations between *HOXB13* status and molecular subtypes, Though it is likely that these associations are driven by *HOXB13* status itself, it is possible that some results are confounded by patient ancestry. *HOXB13* G84E is more common among patients with Nordic ancestry, thus it remains formally possible that Nordic ancestry itself is associated with a difference in prevalence in ETS and PTEN molecular subtypes. However, a major decrease in the prevalence of *ERG* rearrangement and PTEN loss has not be observed in prior studies of Nordic populations compared to unselected European-ancestry patients [48–50].

In conclusion, to our knowledge, this is the first study to rigorously compare the prevalence of the most common molecular subtypes of primary prostate cancer between *HOXB13* G84E carriers and non-carriers, matched for relevant clinical-pathologic variables. We report that the frequency of ETS expression (reflecting underlying *ETS* gene rearrangements) as well as PTEN loss (reflecting underlying *PTEN* deletion) is significantly less common among G84E carriers compared to matched controls. The low rate of ETS family member activation suggests that other genes and/or pathways may play a more important role in driving carcinogenesis in men with G84E mutations. Only further basic investigation will shed light on this possibility and the mechanism by which mutated HOXB13 may contribute to this process. Furthermore, the fact that PTEN loss is less common among G84E carriers raises the question of whether clinical outcomes or overall copy number alteration burden may be different in this population compared to grade and race-matched controls. To answer these questions, additional studies will be required for further molecular subtyping of these tumor cohorts with controls and clinical follow-up.

## MATERIALS AND METHODS

### Patients and tissue samples

With institutional review board approval, a total of 106 heterozygous carriers of HOXB13 *G84E* were identified among patients who underwent radical prostatectomy (RP) for prostate cancer between 1985 and 2011 at the Johns Hopkins Hospital. Genotyping of germline DNA prepared from whole blood or non-cancerous seminal vesicles in this cohort was performed as described in a previous study where clinical and pathologic data from a subset of these cases was reported [3, 34]. Tissue microarrays (TMAs) were prepared from G84E carrier cases using four-fold redundancy of sampling of the index (highest grade/largest size) tumor as well as benign prostate tissue from the same patient. Of these cases, 101 (95%) had adequate tumor tissue present on the TMA for evaluation. Only 78 patients (74%) had complete tissue samples from the radical prostatectomy available to determine index tumor location. Of these, 33% (26/78) of patients had index tumors centered anterior to the urethra (anterior zone tumors). The remainder had posterior index tumors. HOXB13 carrier status was not associated with any clear enrichment of known morphologic subtypes of prostate tumors (eg, ductal, mucinous, pseudohyperplastic etc).

Control cases undergoing RP at the Johns Hopkins Hospital who did not carry G84E were selected for comparison. Because *HOXB13* G84E occurs almost exclusively in patients of European ancestry [3], to procure controls we used stratified random sampling based on patient age and tumor grade to select controls of self-identified European-American (white race) ancestry from previous studies from our institution where cases were not selected for oncologic outcomes. Three TMA cohorts were utilized to glean controls. TMA 1 was derived specifically to compare prostate tumors from African-American (AA) and European-American (EA) subjects [22]. For this TMA, grade-matched EA (*n* = 124) and AA subjects were selected among all men with available tissue and clinical follow-up who underwent RP from 1995 through 2005. Only the EA patients were utilized as controls in the current study. TMA 2 was a similar design: grade-matched Gleason 6 and Gleason 7 prostate tumors from EA (*n* = 59) and AA subjects from 2000–2010. Again, only the European-American patients were utilized as controls in the current study. TMA 3 included 340 consecutive RPs from 2000–2004 not included in the previous microarrays and enriching for subjects with Gleason score > 6, and from these we selected only subjects of EA descent (self-identified white race) [22]. From these three TMAs, a total of 523 EA controls were selected. Of these, 375 (72%) had available *HOXB13* G84E germline genetic status and were known non-carriers (Supplementary Table 1).

Because the prevalence of PTEN loss varies dramatically by pathologic Gleason score and because *HOXB13* G84E carriers tend to be younger at time of radical prostatectomy [34], from the 375 cases, 99 were selected by stratified random sampling by age and Gleason score to be matched to the 101 G84E carriers with complete clinical-pathologic data and PTEN/ERG status.

### Immunohistochemistry (IHC) and interpretation

PTEN and ERG IHC were performed on all TMAs using automated assays as previously described [26, 27, 29]. We have previously published that these assays are highly sensitive and specific for the presence of underlying *PTEN* gene deletion and *ERG* gene rearrangement by gold-standard fluorescence *in situ* hybridization FISH [46, 47]. Briefly, the PTEN protocol uses the Ventana automated staining platform (Ventana Discovery Ultra, Ventana Medical Systems, Tucson, AZ) and a rabbit anti-human PTEN antibody (Clone D4.3 XP; Cell Signaling Technologies, Danvers, MA). The assay was blindly scored by a urologic pathologist (TLL) using a genetically validated scoring system [26, 27, 29]. A tumor biopsy was considered to have PTEN protein loss if the intensity of cytoplasmic and nuclear staining for PTEN was markedly decreased or entirely negative across > 10% of tumor cells compared to surrounding benign glands and/or stroma, which provide internal positive controls for PTEN protein expression. If the tumor showed PTEN protein expressed in > 90% of sampled tumor glands, the tumor was scored as PTEN intact. If PTEN was lost in < 100% of the tumor cells sampled in a given core, the core was annotated as showing heterogeneous PTEN loss in some, but not all, cancer glands (focal loss). Alternatively, if the core showed PTEN loss in 100% of sampled tumor glands, the core was annotated as showing homogeneous PTEN loss. Finally, a small percentage of cores were scored as having ambiguous PTEN IHC results. This occurred when the intensity of the tumor cell staining was light or absent in the absence of evaluable internal benign glands or stromal staining.

ERG immunohistochemistry was performed on the Ventana Benchmark autostaining system using a rabbit monoclonal antibody (EPR 3864) after antigen retrieval in CC1 buffer followed by detection with the Optiview HRP system (Roche/Ventana Medical Systems, Tucson, AZ). Each TMA spot containing tumor was visually dichotomously scored for presence or absence of nuclear ERG signal by a urologic pathologist blinded to the gene expression data (TLL). A spot was considered to be ERG-positive if any tumor nuclei showed ERG positivity, utilizing endothelial cells as an internal positive control in all cases. A tumor was considered ERG positive if all sampled spots were scored as ERG positive, and as ERG negative if all sampled spots were scored as ERG negative.

Immunohistochemistry for SPINK1 utilized the mouse monoclonal anti-SPINK1 antibody in a manual staining protocol after citrate antigen retrieval (ab58227, 1:250, Abcam, Cambridge, UK) with the UltraVision Quanto secondary reagent kit according to the manufacturer's directions (Thermo Fisher Scientific, Waltham, MA). SPINK1 IHC was scored as positive if any cells showed positive cytoplasmic immunostaining in one or more cores sampled from the tumor on the TMA.

p53 IHC was performed on the Ventana Benchmark autostaining system using a mouse monoclonal antibody (BP53-11) after antigen retrieval in CC1 buffer followed by detection with the iView HRP system (Roche/Ventana Medical Systems, Tucson, AZ). Each tissue microarray spot containing tumor cells was visually dichotomously scored for presence or absence of nuclear p53 accumulation by a urologic pathologist (TLL). A spot was considered to show p53 nuclear accumulation if > 10% of tumor nuclei showed p53 positivity. A tumor was considered to show p53 nuclear accumulation if any sampled spot was scored as p53-positive, and as lacking p53 nuclear accumulation if all sampled spots were scored as p53 negative. Nuclear accumulation of p53 by this assay is more than 90% sensitive and specific for the presence of an underlying missense mutation in *TP53*, which generally stabilize the protein (Guedes et al, in progress) [51]. Notably, this assay does not detect other loss-of-function alteration inTP53, such as frame shift mutations, splice site mutation or homozygous deletions.

HOXB13 immunohistochemistry was performed manually using an affinity-purified polyclonal sheep IgG (AF8156, 1:100, 2ug/ml, R&D Systems, Minneapolis, MN), after citrate antigen retrieval and visualized with a Pierce HRP conjugated rabbit anti sheep IgG (1:1000, Thermo Fisher Scientific, Waltham, MA). The immunogen used for the primary antibody was an E.coli -derived human HOXB13 fragment, amino acid residues 1-102. In western blot, this antibody recognizes a single band of mol wt 37kd in extracts of prostate tissues and cell lines (Supplementary Figure 1).

### Detection of ETV1/4/5 over-expression by *in situ* hybridization

Chromogenic *in situ* hybridization for ETV1/4/5 RNA was performed with the RNAscope® FFPE kit 2.5 from Advanced Cell Diagnostics (ACD, Hayward, CA) following the manufacturer's recommendations. ETV1 (NM_004956), ETV4 (NM_001986.2) and ETV5 (NM_004454.2) probes, validated in a recent study, were utilized [38]. Probes for PPIB (NM_000942.4) were used as positive control. We re-validated the assay in our own laboratory, using cases known by sequencing to be positive for ETV1, 4 or 5 fusions [52], as well as LNCaP (ETV1+) [53], DU145 and CWr22 cell lines. Normal prostate tissues from radical prostatectomy specimens were used as negative control tissue. All cases were qualitatively scored by a blinded surgical pathologist (TLL and AT), using a dichotomous scoring system to assess for positive cases (cases with any distinct red punctae present in any tumor cells in any punch, Figure 1).

### Statistical analysis

Clinical and pathological characteristics were assessed in the study population. Summary statistics provided are median (interquartile range) for continuous variables and number (proportion) for categorical variables. Comparison testing was performed using the Mann-Whitney and chi-squared tests as appropriate. All statistical analysis was performed using Stata Intercooled v13.1 (College Station, TX).

## SUPPLEMENTARY MATERIALS FIGURES AND TABLES


