# Supplementary material for: Somatic molecular subtyping of prostate tumors from HOXB13 G84E carriers
Source: Oncotarget. 2017 Feb 8;8(14):22772–82. doi: 10.18632/oncotarget.15196 (PMC5410261; doi:10.18632/oncotarget.15196)
Supplement: Supplementary file 1 [file oncotarget-08-22772-s001.pdf]

Somatic molecular subtyping of prostate tumors from *HOXB13* G84E carriers

Supplementary Materials

Supplementary Table 1: Clinical-pathologic data for HOXB13 G84E carriers and controls

	HOXB13 **WT (*n* = 375)**	***HOXB13*** **G84E (*n* = 102)**	***P*-value**
Age (median, IQR)	59 (54–63)	55 (51–60)	< 0.001
Prostate weight, g	50 (42–60)	47 (39–54)	0.0027
RP grade group			
1 (GS6)	109 (29.1%)	59 (58.4%)	< 0.001
2 (GS3+4=7)	143 (38.1%)	25 (24.8%)	
3 (GS4+3=7)	79 (21.1%)	9 (8.9%)	
4 (GS8)	32 (8.5%)	3 (3.0%)	
5 (GS9-10)	12 (3.2%)	5 (5.0%)	
RP pathologic stage			
pT2N0	214 (57.1%)	70 (70%)	0.069
pT3aN0	117 (31.2%)	24 (24%)	
pT3bN0	33 (8.8%)	3 (3%)	
pTxN1	11 (2.9%)	3 (3%)	

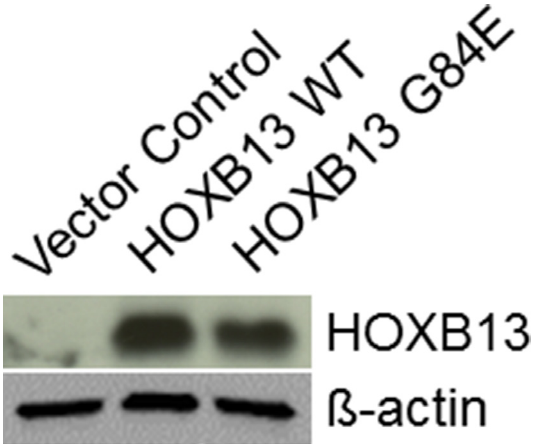

Supplementary Figure 1: Immunoblot using HOXB13 antibody utilized for immunohistochemistry in Figure 1. DU145 cell lines stably expressing *HOXB13* WT or G84E were generated using lentivirus system. Whole cell lysates from cell lines were prepared with sonication in a buffer containing 50 mM Tris-HCl pH 7.5, 1 mM EDTA and 2%SDS. Western blot analysis of 5 ug of protein was performed using 0.2 µg/ml HOXB13 antibody (AF8156, R&D Systems) and ß-actin antibody (ab8227, Abcam). The immunogen for this antibody was an E.coli -derived human HOXB13 fragment, amino acid residues 1-102. On immunoblotting, this antibody recognizes a single band of molecular weight 37 kd in extracts of prostate tissues and cell lines.

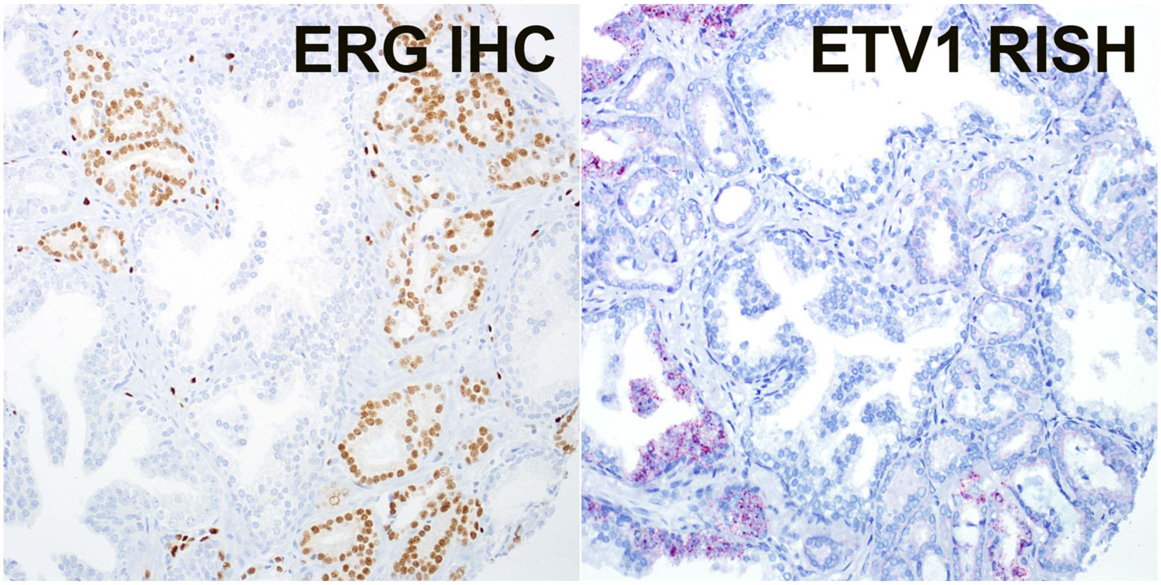

Supplementary Figure 2: Case with heterogeneous staining for ERG by immunohistochemistry (IHC) and ETV1 RNA *in situ* hybridization (RISH). This case appears to be a collision tumor as some tumor glands are positive for ERG and others are positive for ETV1. Images at 200× magnification.
